# Supplementary material for: Correction of dysregulated lipid metabolism normalizes gene expression in oligodendrocytes and prolongs lifespan in female poly-GA C9orf72 mice
Source: Nat Commun. 2025 Apr 11;16:3442. doi: 10.1038/s41467-025-58634-4 (PMC11992041; doi:10.1038/s41467-025-58634-4)
Supplement: Supplementary file 1 — Supplementary Information [file 41467_2025_58634_MOESM1_ESM.pdf]

Supplementary information for:

Correction of dysregulated lipid metabolism normalizes gene expression in oligodendrocytes and prolongs lifespan in female poly-GA C9orf72 mice

Ali Rezaei, Virág Kocsis-Jutka, Zeynep I. Gunes, Qing Zeng, Georg Kislinger, Franz Bauernschmitt, Huseyin Berkcan Isilgan, Laura R. Parisi, Tuğberk Kaya, Sören Franzenburg, Jonas Koppenbrink, Julia Knogler, Thomas Arzberger, Daniel Farny, Brigitte Nuscher, Eszter Katona, Ashutosh Dhingra, Chao Yang, Garyfallia Gouna, Katherine D. LaClair, Aleksandar Janjic, Wolfgang Enard, Qihui Zhou, Nellwyn Hagan, Dimitry Ofengeim, Eduardo Beltrán, Ozgun Gokce, Mikael Simons, Sabine Liebscher, Dieter Edbauer

This PDF file includes Figures S1 to S13.

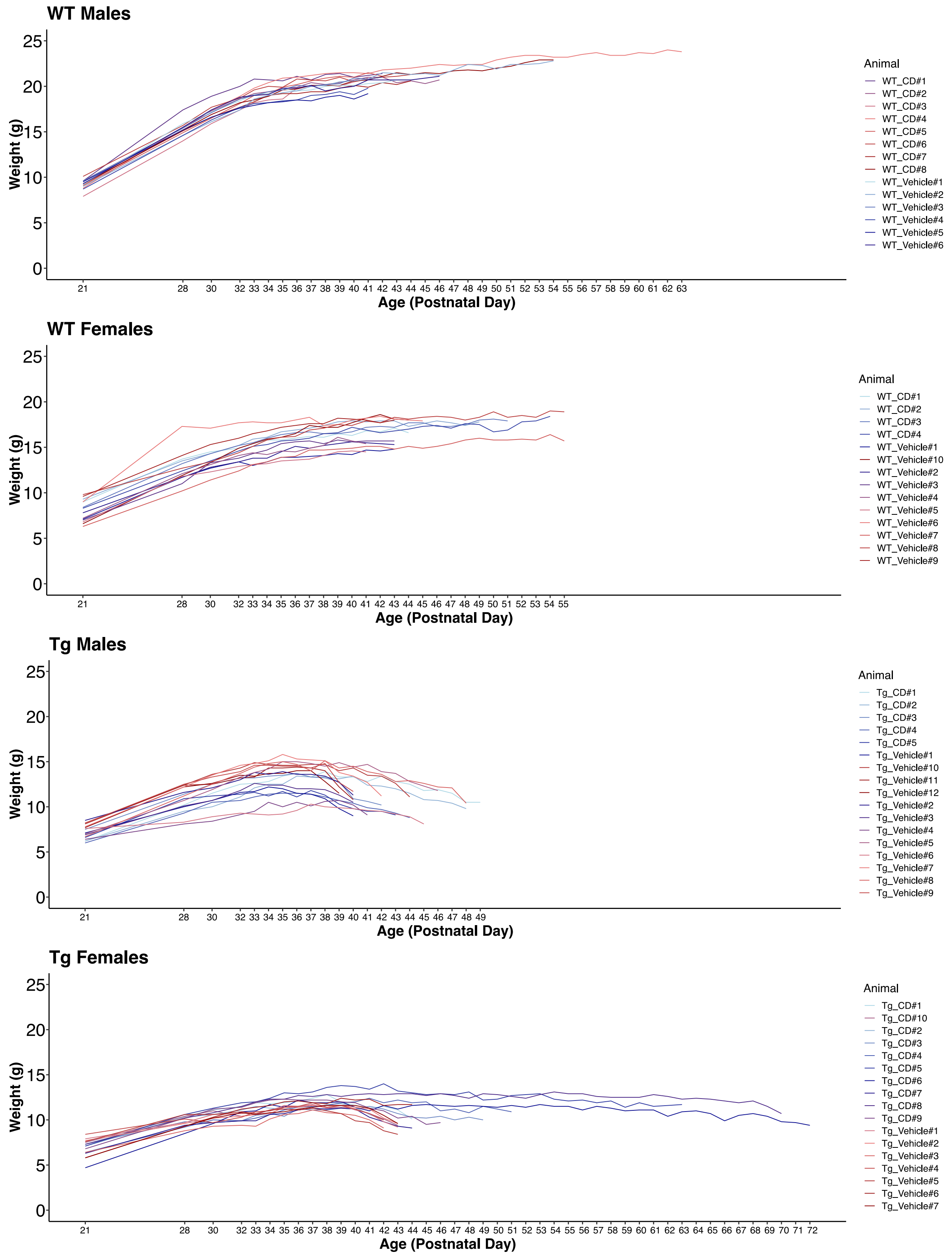

Figure S1 | CD treatment ameliorates the body weight loss in female GA-Nes mice.

Body weights of GA-Nes and wild-type littermates from the survival study in Figure 1b-c are plotted on P21, P28, P30, P32 and every day after P32 until euthanasia. CD does not appear to affect body weight in either wild-type or male transgenic animals. In female GA-Nes animals, the rate of body weight loss is slowed, resulting in longer survival, as the most common reason for euthanasia in GA-Nes mice is a body weight loss of 20% below the maximum weight.

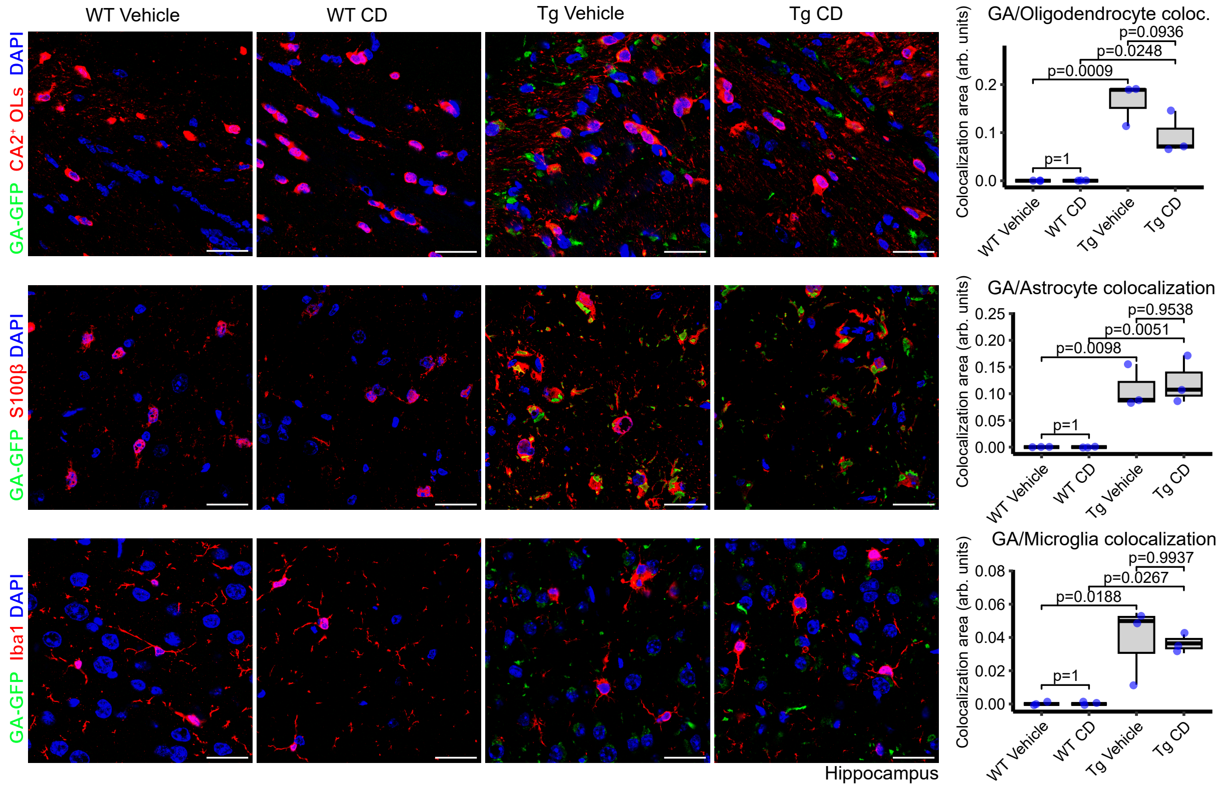

Figure S2 | GFP-(GA)$_{149}$ is widely expressed in oligodendrocytes and astrocytes in GA-Nes mice.

Immunofluorescence analysis of glial marker proteins and endogenous GFP-(GA)$_{149}$ fluorescence and quantification of colocalization area. Transgene expression is widespread in oligodendrocytes and astrocytes, but CD does not significantly affect colocalization. In microglia, colocalization of GFP-(GA)$_{149}$ is much less compared to the other glial subtypes and CD has no significant effect. n=3 in each of the four conditions, from independent biological replicates (ANOVA with Tukey's HSD posthoc test).

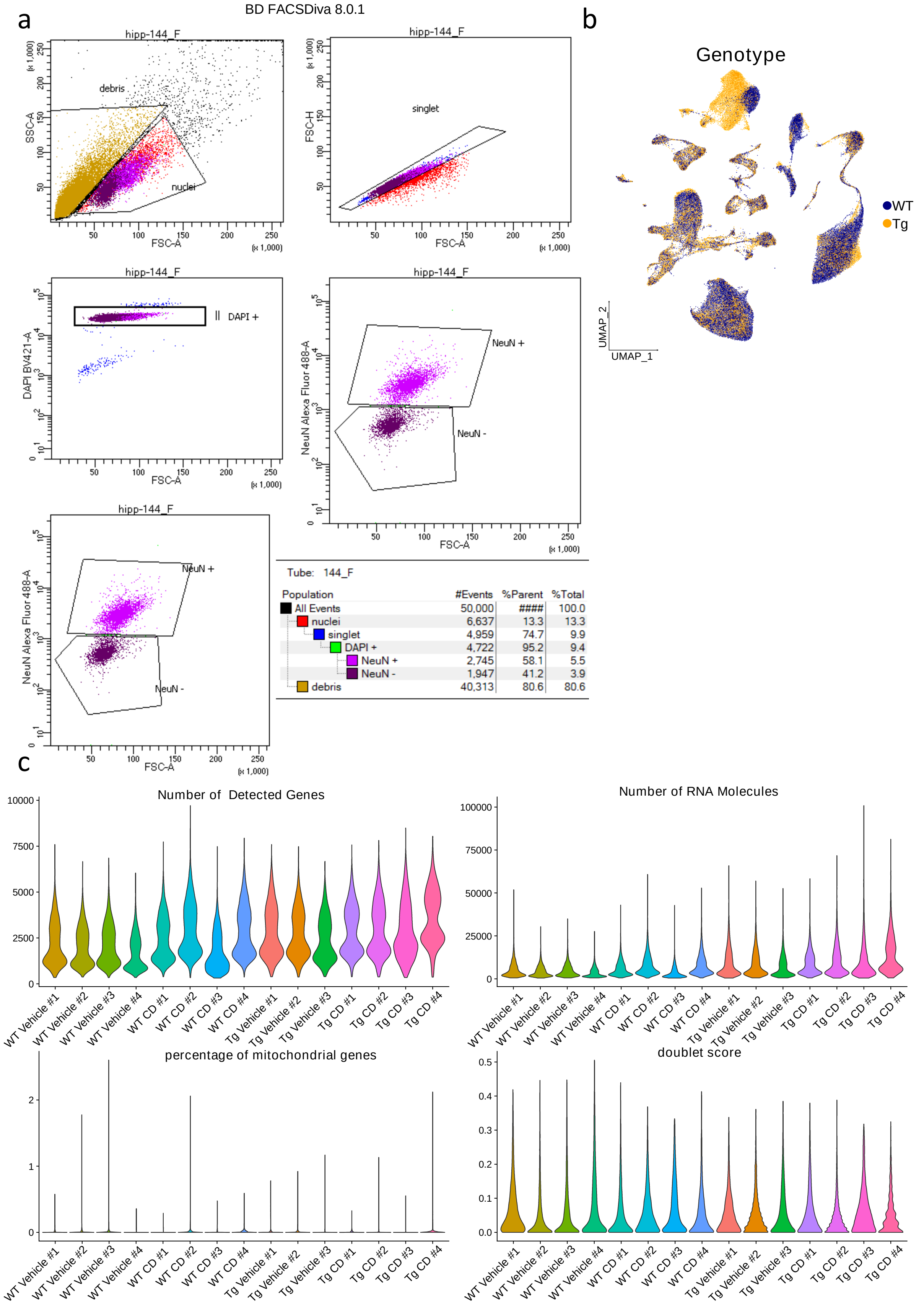

Figure S3 | GA-Nes FANS and snRNAseq QC.

a) An example of the Fluorescence-Activated Nuclei Sorting (FANS) pipeline applied to each sample is depicted. 10,000 DAPI+ nuclei are sorted into 5000 NeuN$^+$ and NeuN$^-$ events. **b)** UMAP with shuffled points (to avoid complete overlap in certain clusters) highlights the effect of transgene expression on microglia and oligodendrocytes in GA-Nes mice. **c)** QC measurements for all 15 animals in snRNAseq show similar and good quality across all samples.

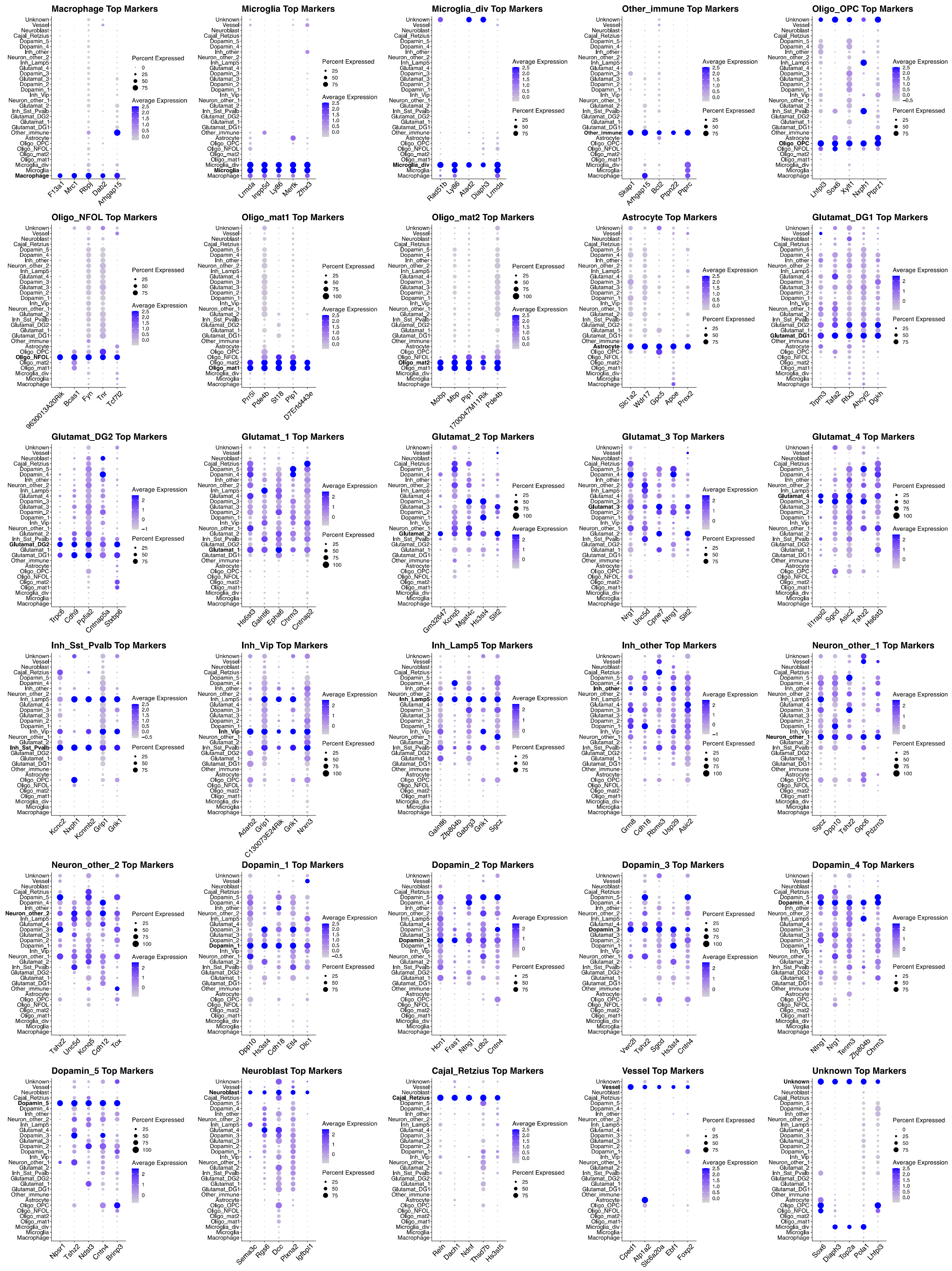

Figure S4 | Top marker expression in each cluster across all clusters of GA-Nes mice.

DotPlot of expression of the top 5 markers (generated by Seurat) in each cluster is depicted across all clusters in GA-Nes mice.

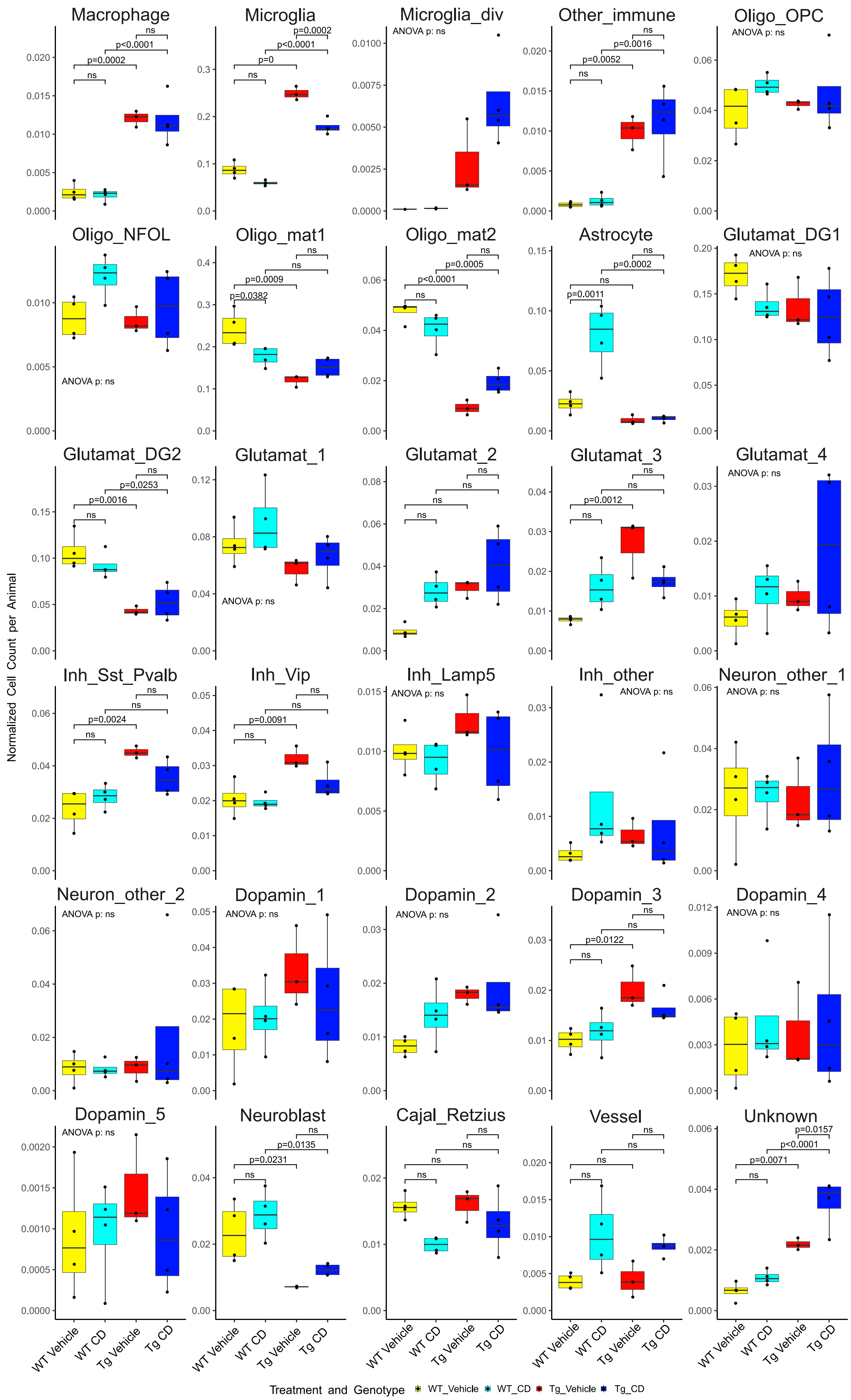

Figure S5 | Immune cells are upregulated in GA-Nes mice.

Normalized number of nuclei from snRNAseq in each cluster per each animal for each of the four experimental conditions is normalized to the total number of detected nuclei from the respective animal. This number is then compared across the four conditions (ANOVA with Tukey's HSD; n: WT Vehicle=4, WT CD=4, Tg Vehicle=3, Tg CD=4).

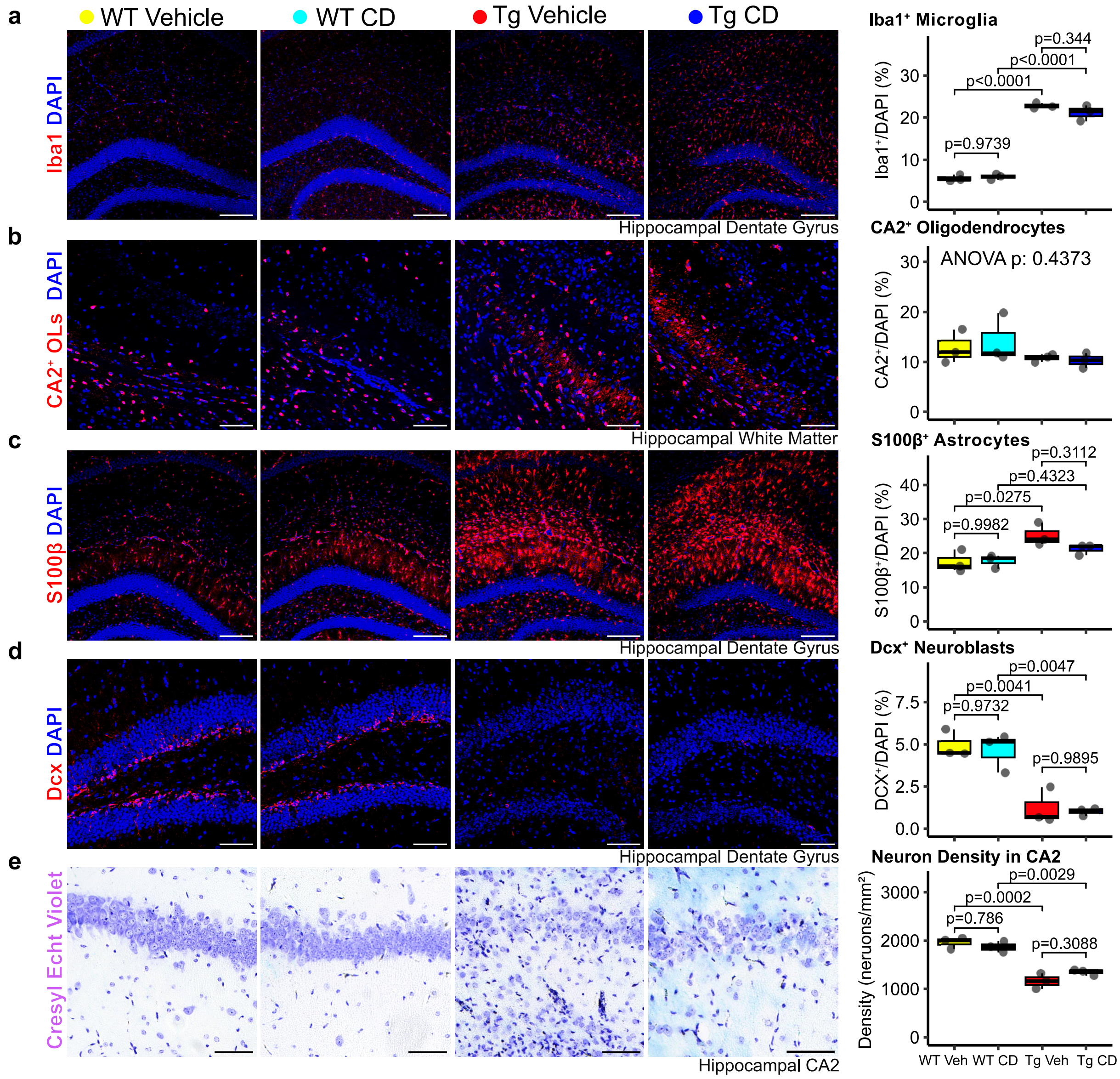

Figure S6 | Cell density at P40 is affected by transgene expression but not by CD in GA-Nes mice.

Analysis of glial and neuronal cell counts using immunofluorescence and cresyl violet staining in P40 GA-Nes mice treated with CD or vehicle as in Figure 1. Quantitative analysis from hippocampal regions as indicated using n=3 independent biological replicates in each condition (ANOVA, post hoc by Tukey's HSD). **a)** GA-Nes mice show increased Iba1^{+} microglia numbers, but CD treatment has no effect. **b)** CA2^{+} oligodendrocyte density is not significantly affected by transgene expression or CD treatment. **c)** S100β^{+} astrocyte density is increased in GA-Nes mice, but CD injection does not significantly change astrocyte density. **d)** Dcx^{+} neuroblasts are significantly reduced with transgene expression, and CD does not rescue this loss. **e)** Neuron density in the hippocampal region of the CA2 hippocampus is significantly reduced, but CD injection does not rescue the neuron loss.

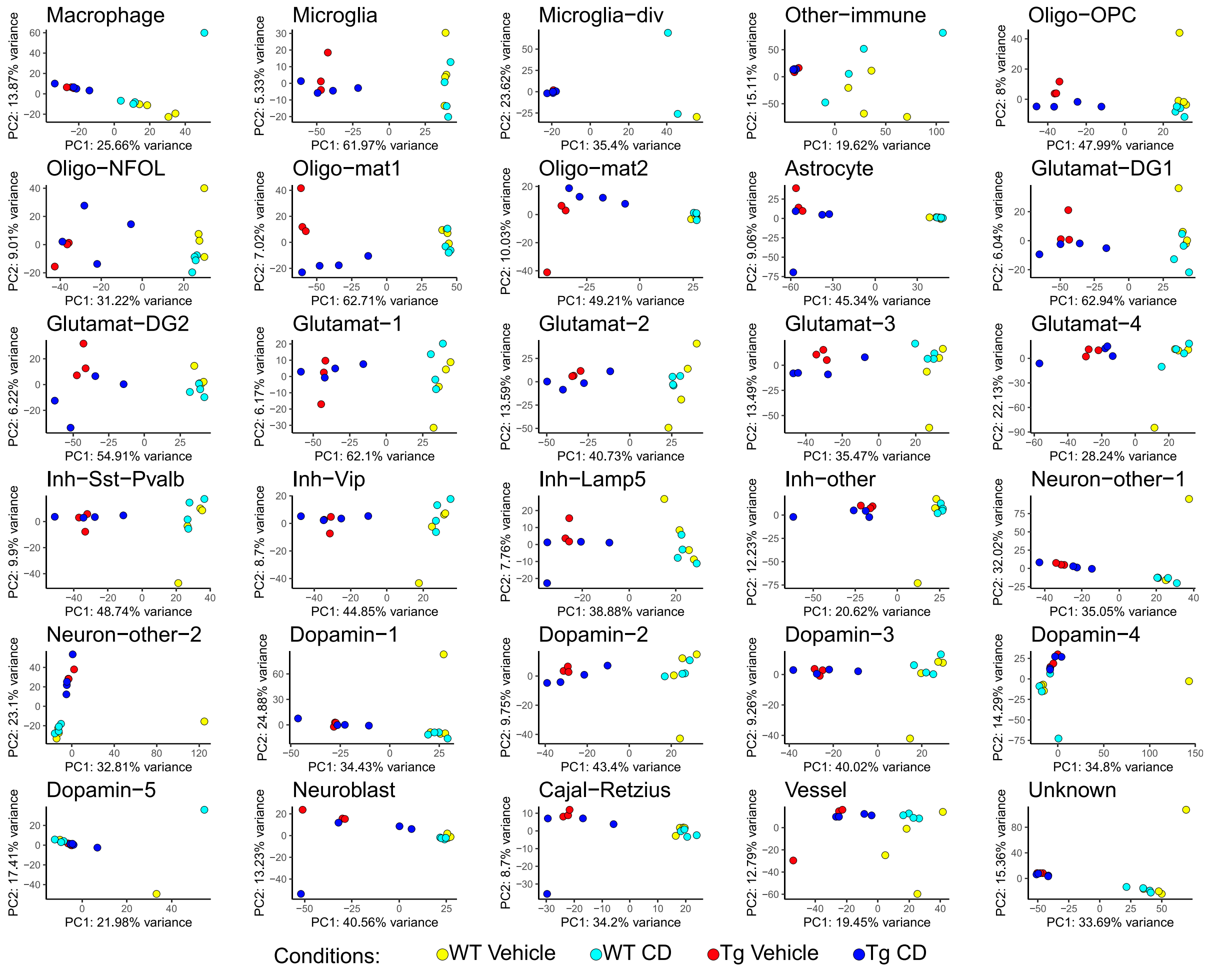

Figure S7 | Principal component analysis within each cluster separates wildtype and transgenic animals.

Pseudobulk analysis is performed for each cluster, and a PCA is performed on all expressed genes across animals in the four conditions. The PCA clearly separates the transgenic animals from wildtype animals across almost all clusters. CD treatment on transgenic mice is most clearly shown in mature oligodendrocyte clusters and neuroblast clusters (n: WT Vehicle=4, WT CD=4, Tg Vehicle=3, Tg CD=4).

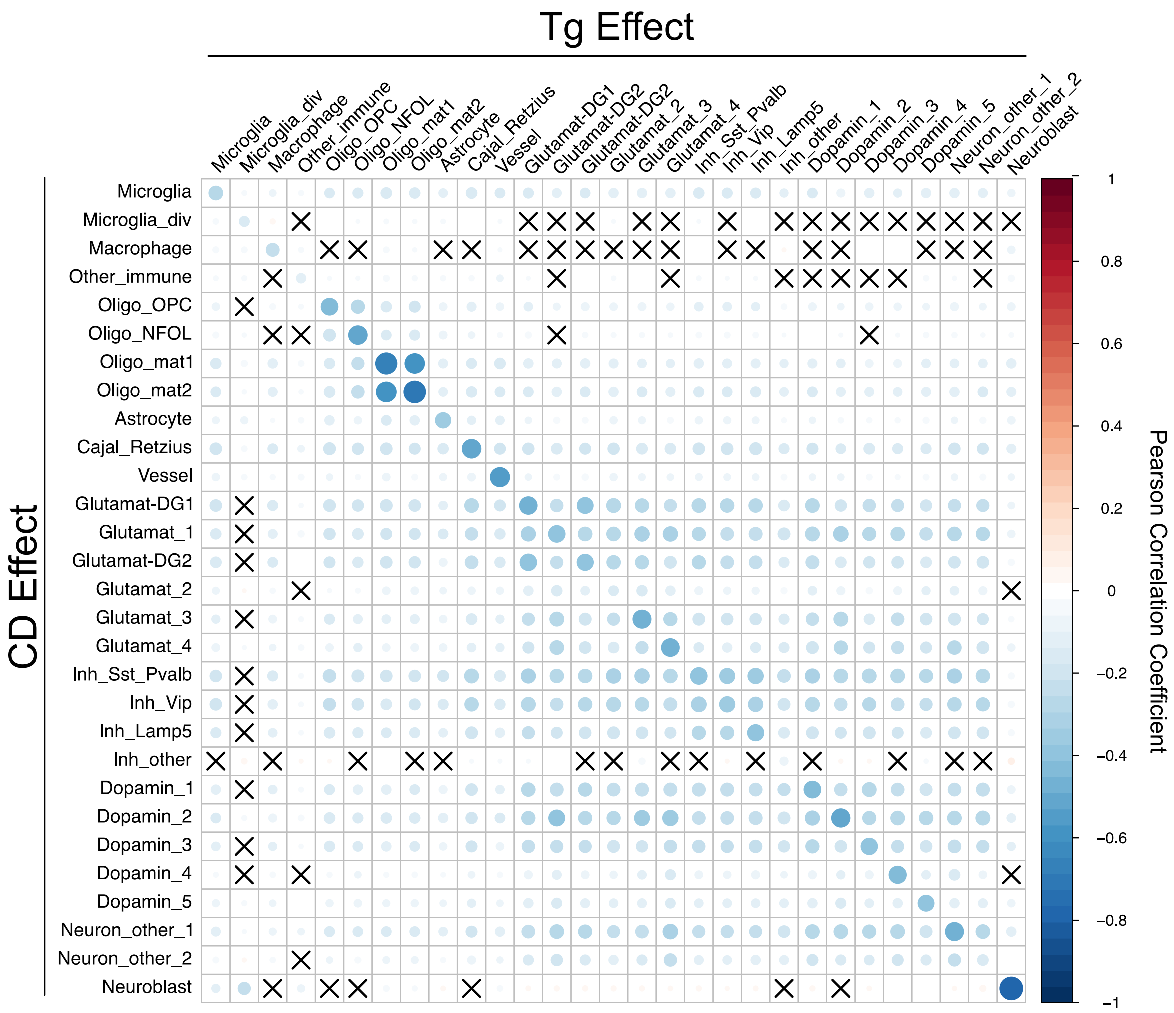

Figure S8 | Main targets of CD administration are oligodendrocytes and neuroblasts.

Correlation plot depicts partial CD rescue effect across various clusters, with the most obvious effect being observed in oligodendrocytes and neuroblast clusters. Color indicates Pearson's correlation coefficient. Crossed out comparisons are not significantly correlated.

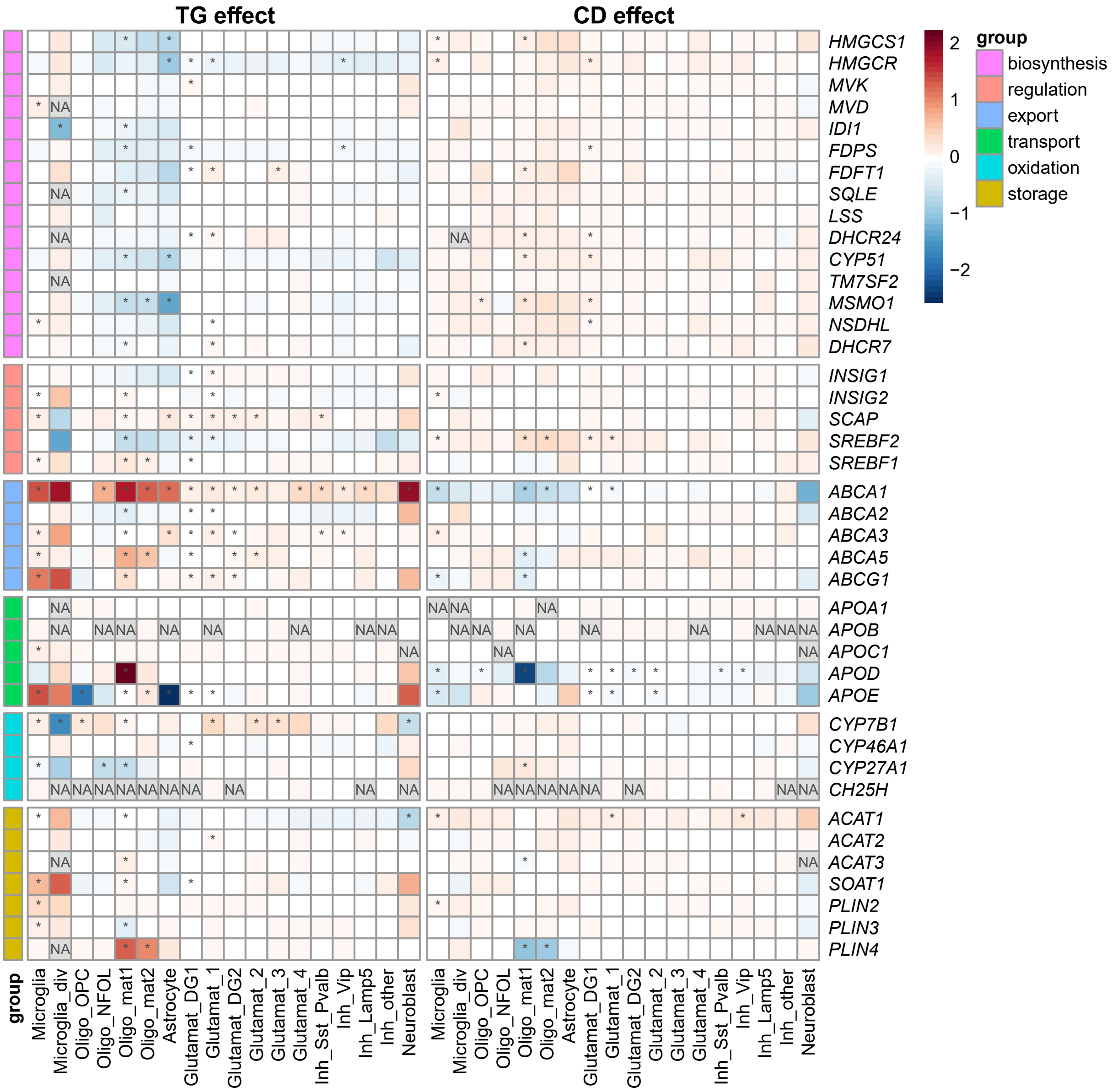

Figure S9 | Cholesterol dysmetabolism across different cell clusters in GA-Nes mice

Pseudobulk analysis of snRNAseq data from Figure 2 for the indicated cell clusters, plotting log_2-fold change between TG vehicle vs. WT vehicle (transgene effect; n=3 Tg Vehicle vs n=4 WT Vehicle) and TG CD vs. TG vehicle (CD effect; n=4 Tg CD vs n=3 Tg Vehicle). While there is a general decrease in cholesterol biosynthesis and an increase in export and storage genes in different cell types in GA-Nes mice, CD treatment mainly affected the oligodendrocyte clusters (especially Oligo_mat1). Asterisks indicate significant changes in DESeq2 analysis (adjusted $p < 0.05$).

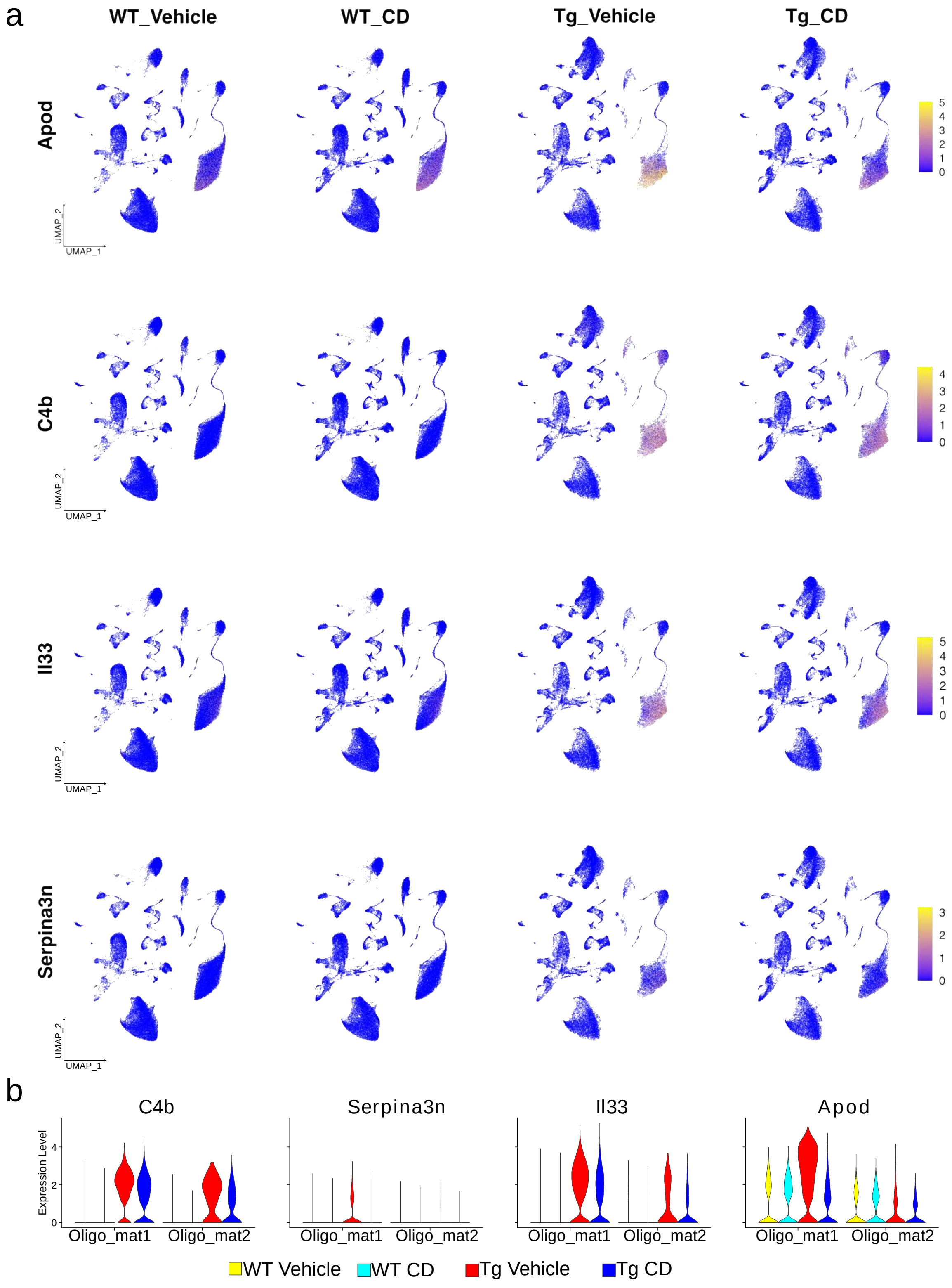

Figure S10 | CD administration partially reverts expression of DOL markers.

Our top DOL markers, which overlap with previously published markers, are negligibly expressed in wildtype conditions but highly upregulated in Tg Vehicle condition. Administration of CD (Tg CD condition) partially reverses this upregulation as shown in in FeaturePlots (a) and Violin Plots (b). FeaturePlots also clearly indicate that the expression of the DOL markers Serpina3n, Il33, Apod, C4b is mainly in oligodendrocytes. (n: WT Vehicle=4, WT CD=4, Tg Vehicle=3, Tg CD=4).

MSD Cytokine Panel

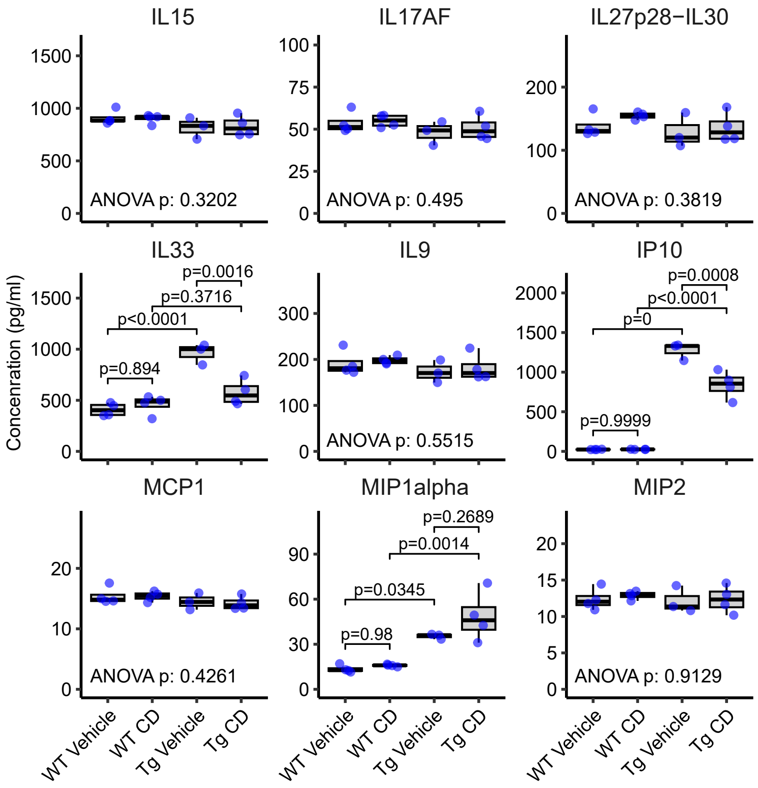

Figure S11 | Cytokine response in GA-Nes mice treated with CD.

Immunoassay with MSD cytokine panel from hindbrain samples show upregulation of IL33, IP10, and MIP1alpha due to the transgene effect, while CD completely rescues IL33 and partially rescues IP10. ANOVA statistics with Tukey's HSD post hoc (n: WT Vehicle=4, Tg Vehicle=3, WT CD=4, Tg CD=4).

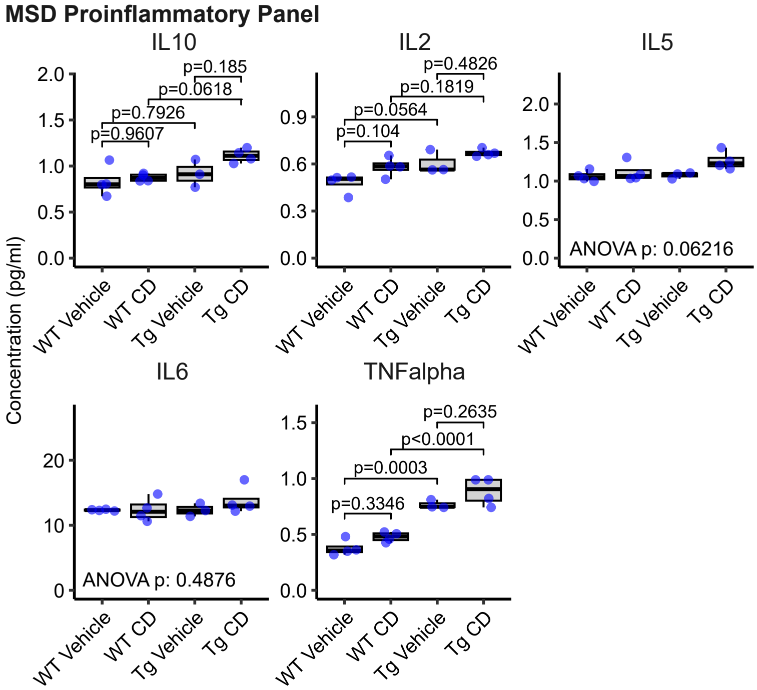

Figure S12 | Proinflammatory response in GA-Nes mice treated with CD.

Immunoassay with MSD proinflammatory panel shows upregulation of TNFα but no rescue with CD. ANOVA statistics with Tukey's HSD (n: WT Vehicle=4, Tg Vehicle=3, WT CD=4, Tg CD=4).

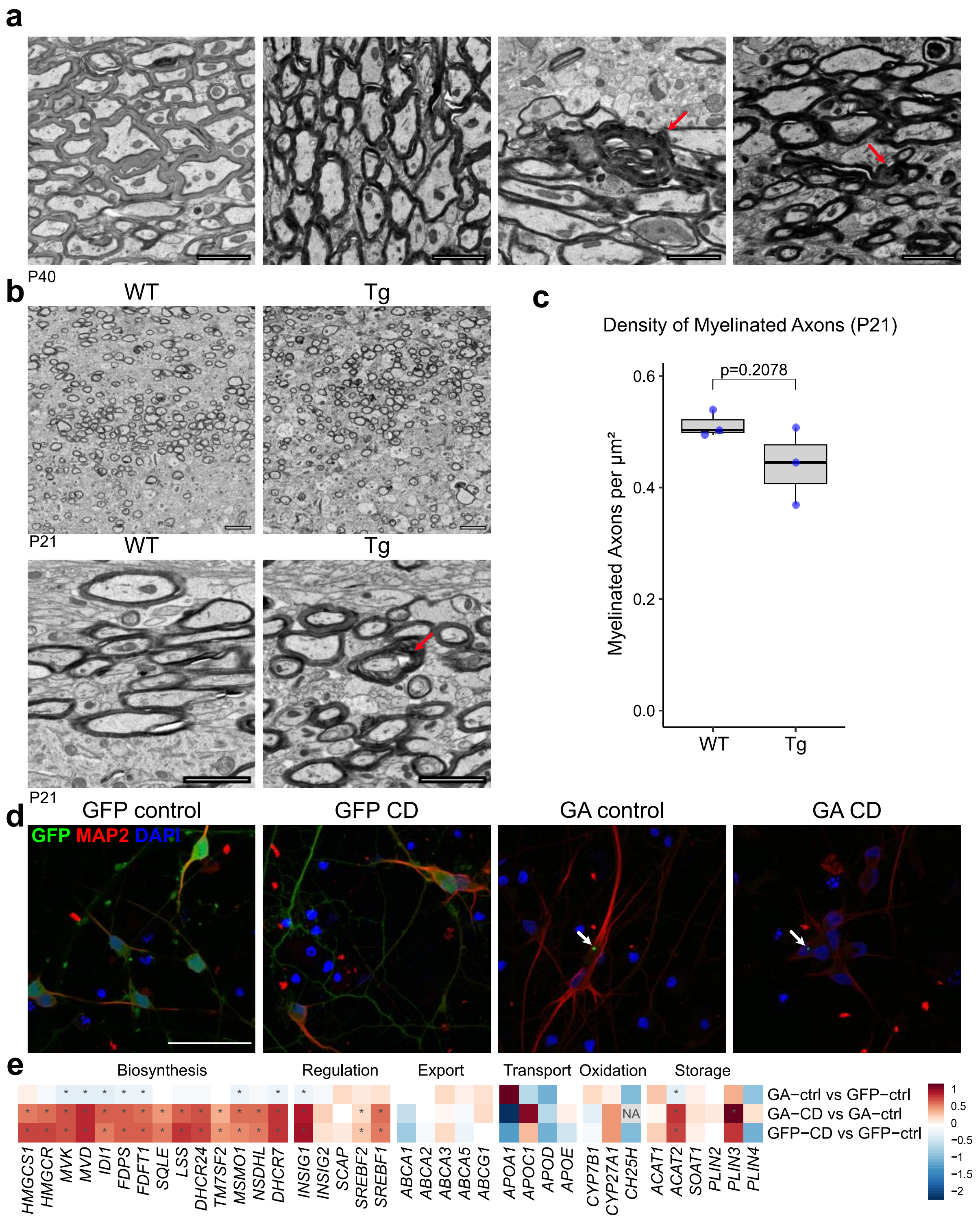

Figure S13 | Myelination is not affected in GA-Nes mice at P21. Poly-GA has minimal effects on neuronal cholesterol metabolism.

a) High magnification electron microscopy analysis of myelination as in Figure 4d/e. Swollen axons with organelle accumulation are detect in GA-Nes mice at P40 (red arrow) in both Tg Vehicle and Tg CD conditions. Scale bars = 2 µm. **b, c)** Electron microscopy analysis in the corpus callosum of GA-Nes mice at P21. The density of myelinated axons in corpus callosum is, despite a trend, not significantly different between transgenic and non-transgenic mice. Scale bars = 3 µm for upper two images and 2 µm for bottom two images. **d, e)** iPSC-derived neurons transduced with $(GA)_{149}$-GFP and GFP lentiviral constructs and treated with CD. Immunofluorescence shows expression of diffuse GFP and aggregated $(GA)_{149}$-GFP in MAP2-positive neurons (scale bar 50 µm). Bulk RNAseq shows some limited but significant effects of $(GA)_{149}$-GFP on neuronal cholesterol biosynthesis, while CD treatment (2.5 mg/mL from day 10 to day 19 after differentiation) significantly increased cholesterol biosynthesis in poly-GA and control neurons (n: GFP-ctrl=3, GFP-CD=4, GA-ctrl=4, GA-CD=4).

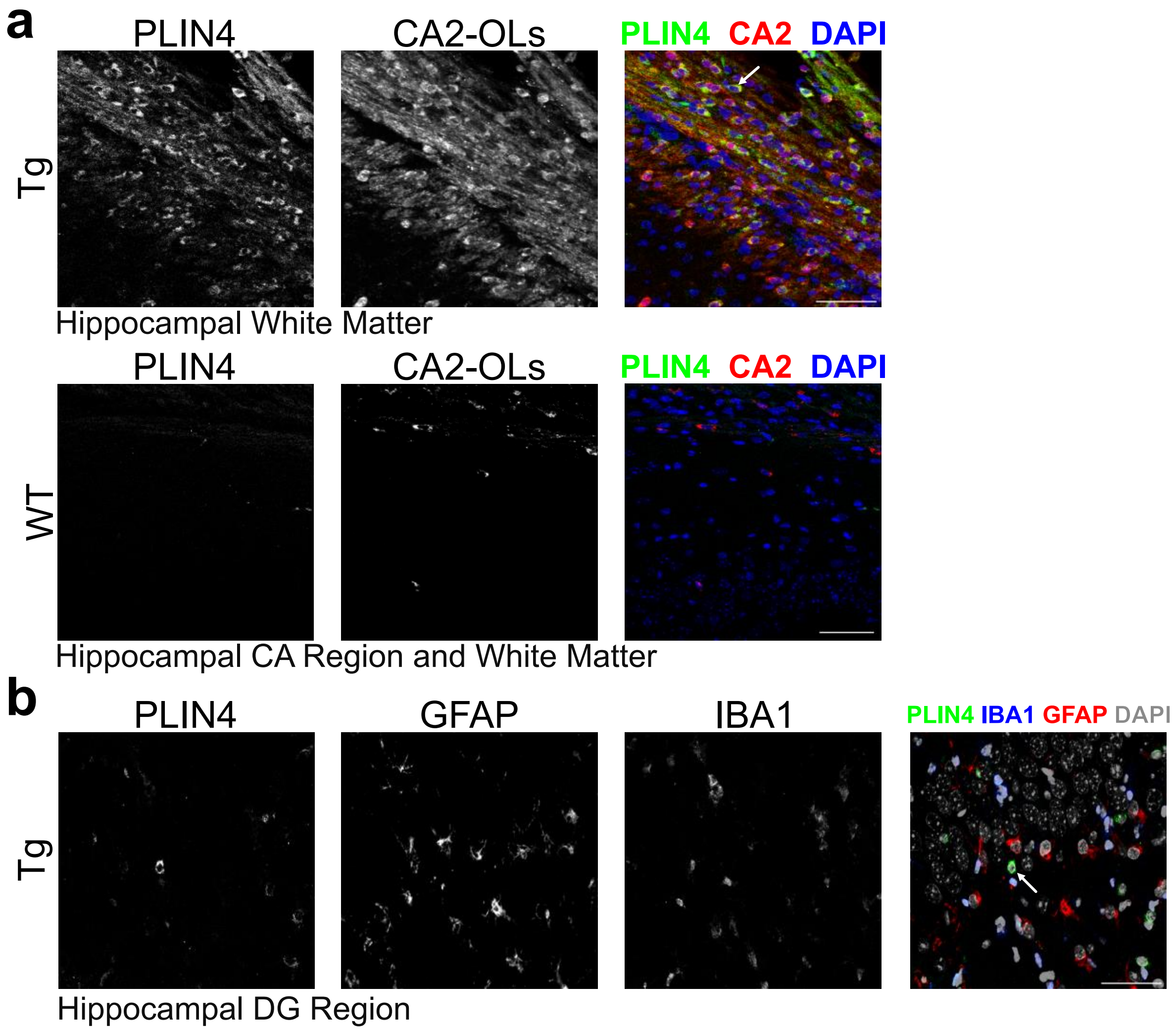

Figure S14 | PLIN4 in transgenic animals colocalizes with oligodendrocytes but not other glia.

a) PLIN4 colocalization is confirmed in CA2+ oligodendrocytes. White arrow points to an example PLIN4+ cell. Plin4 is not detected in wildtype mice. Images are representative of four independent experiments. Shown images are adjusted for brightness and contrast (scale bars = 50 µm). **b)** PLIN4 does not colocalize with Iba1+ microglia or GFAP+ astrocytes (scale bar = 50 µm). White arrow points to an example PLIN4+ cell. Images are representative of two independent experiments. Shown images are adjusted for brightness and contrast.
