# Supplementary material for: Correction of dysregulated lipid metabolism normalizes gene expression in oligodendrocytes and prolongs lifespan in female poly-GA C9orf72 mice
Source: Nat Commun. 2025 Apr 11;16:3442. doi: 10.1038/s41467-025-58634-4 (PMC11992041; doi:10.1038/s41467-025-58634-4)
Supplement: Supplementary file 2 — Description of Additional Supplementary Files [file 41467_2025_58634_MOESM2_ESM.pdf]

Description of Additional Supplementary Files

File name: Supplementary Data 1
Description: Absolute Cell Numbers in Clusters of GA-Nes snRNAseq Data

File name: Supplementary Data 2
Description: Euclidian Distances in PCAs of GA-Nes snRNAseq Data

File name: Supplementary Data 3
Description: Differential Gene Expression Table of GA-Nes snRNAseq Data

File name: Supplementary Data 4
Description: Differential Gene Expression Table of iPSC RNAseq Data
